# The Selective Estrogen Receptor Modulator Raloxifene Inhibits Neutrophil Extracellular Trap Formation

**DOI:** 10.3389/fimmu.2016.00566

**Published:** 2016-12-07

**Authors:** Roxana Flores, Simon Döhrmann, Christina Schaal, Abdul Hakkim, Victor Nizet, Ross Corriden

**Affiliations:** ^1^Department of Pediatrics, Division of Host-Microbe Systems and Therapeutics, University of California San Diego, La Jolla, CA, USA; ^2^Department of Physiological Chemistry, University for Veterinary Medicine Hannover, Hanover, Germany; ^3^Department of Molecular Biology, Massachusetts General Hospital, Harvard Medical School, Boston, MA, USA; ^4^Department of Pharmacology, University of California San Diego, La Jolla, CA, USA

**Keywords:** neutrophils, raloxifene, MRSA, host–pathogen interactions, extracellular traps

## Abstract

Raloxifene is a selective estrogen receptor modulator typically prescribed for the prevention/treatment of osteoporosis in postmenopausal women. Although raloxifene is known to have anti-inflammatory properties, its effects on human neutrophils, the primary phagocytic leukocytes of the immune system, remain poorly understood. Here, through a screen of pharmacologically active small molecules, we find that raloxifene prevents neutrophil cell death in response to the classical activator phorbol 12-myristate 13-acetate (PMA), a compound known to induce formation of DNA-based neutrophil extracellular traps (NETs). Inhibition of PMA-induced NET production by raloxifene was confirmed using quantitative and imaging-based assays. Human neutrophils from both male and female donors express the nuclear estrogen receptors ERα and ERβ, known targets of raloxifene. Similar to raloxifene, selective antagonists of these receptors inhibit PMA-induced NET production. Furthermore, raloxifene inhibited PMA-induced ERK phosphorylation, but not reactive oxygen species production, pathways known to be key modulators of NET production. Finally, we found that raloxifene inhibited PMA-induced, NET-based killing of the leading human bacterial pathogen, methicillin-resistant *Staphylococcus aureus*. Our results reveal that raloxifene is a potent modulator of neutrophil function and NET production.

## Introduction

Neutrophil extracellular traps (NETs), first described in 2004 (1), are DNA-based structures decorated with antibacterial components (e.g., histones, antimicrobial peptides, and myeloperoxidase) that form during a specialized cell death pathway (NETosis) to ensnare and kill pathogens. Since their initial discovery, NETs have been shown to play an important role in host defense; however, the molecular mechanisms that control NET production remain a topic of active investigation. Although NET production is typically driven in concert with the generation of reactive oxygen species (ROS) and activation of PKC, additional pathways that facilitate NET production (e.g., ERK activation, histone citrullination, and intracellular ceramide accumulation) have been identified in recent years (2–4). Identification of novel pathways that modulate NETosis could reveal new therapeutic targets to enhance antibacterial innate immunity or inhibit aberrant neutrophil-mediated inflammatory responses.

Recently, we demonstrated that tamoxifen, an estrogen receptor modulator classically described as an estrogen receptor antagonist, is a potent NET inducer (4). Our results indicated that tamoxifen-induced NET production is largely driven by an accumulation of intracellular ceramide resulting from inhibition of glucosylceramide synthase. This finding prompted us to investigate whether other selective estrogen receptor modulators (SERMs) with pharmacological properties distinct from tamoxifen may also modulate neutrophil function/NET production. Of immediate interest was raloxifene, an FDA-approved compound typically prescribed for the prevention or treatment of osteoporosis in postmenopausal women (5). Similar to tamoxifen, the most well-characterized pharmacological targets of raloxifene are the nuclear estrogen receptors ERα and ERβ (6). Although raloxifene has been described to exhibit potent anti-inflammatory effects (7), our understanding of how it affects neutrophil function remains limited (8), with no studies having directly assessed its effects on human neutrophils. Intriguingly, however, raloxifene treatment reduces plasma neutrophil myeloperoxidase levels *in vivo* (9). Because myeloperoxidase is released into the plasma following NET induction (10), this association suggested that raloxifene may inhibit NET production. Here, we provide evidence that raloxifene prevents neutrophil cell death in response to treatment with the NET inducer phorbol 12-myristate 13-acetate (PMA). Building on these results, we use both quantitative and imaging-based approaches to define the effects of raloxifene on NET production and probe the molecular mechanisms underlying this effect.

## Materials and Methods

### Materials

Raloxifene hydrochloride, MPP dihydrochloride, and PHTTP were purchased from Tocris Bioscience (Bristol, UK). PMA, micrococcal nuclease from *Staphylococcus aureus*, the small molecule library used for screening and all other compounds were obtained from Sigma-Aldrich (St. Louis, MO, USA).

### Cell Viability Screen

An image-based screen, described in detail in Ref. (2), was used in conjunction with the Sigma LOPAC (Library of Pharmacologically Active Compounds; Sigma-Aldrich, St. Louis, MO, USA) library of ~1280 pharmacologically active small molecules. Following exposure to compounds from this library (each at a final concentration of 100 μM), cell viability in response to 4-h PMA treatment was quantified by fixing cells with 2% paraformaldehyde, staining nuclei with the fluorescent DNA stain Sytox Green (2 μM; Life Technologies, Carlsbad, CA, USA), and imaging using an Ascent Fluoroskan MTP reader (Thermo Scientific).

### Neutrophil Isolation

Venous blood was collected from healthy volunteers according to an approved protocol. Heparin was used as an anticoagulant. Neutrophils were isolated using Polymorphprep density gradient medium (Axis-Shield, Dundee, Scotland) according to the manufacturer’s protocol.

### Fluorescence Microscopy/NET Visualization

To visualize NETs, cells were seeded in Nunc Lab-Tek II Chambered Coverglass slides (Thermo Fisher, Waltham, MA, USA) at a density of 2 × 10^5^ cells/well. Following incubation with indicated antagonist compounds, NET production was induced *via* addition of 25 nM PMA prior to a 2-h incubation at 37°C with 5% CO_2_. Cells were fixed by addition of paraformaldehyde (4% final) for 10 min at 24°C. Following three washes with PBS, neutrophils were permeabilized *via* incubation in Triton X-100 solution (0.1%) for 10 min at 24°C. After an additional three washes with PBS, DNA was stained with 2 μM Sytox Green (Life Technologies, Carlsbad, CA, USA) for 10 min at 24°C; slides were then washed a final three times with PBS prior to imaging using a Zeiss AxioObserver D1 microscope equipped with an LD A-Plan 20X/0.35 Ph1 objective (Carl Zeiss AG, Oberkochen, Germany).

Imaging of intracellular ceramide was performed by permeabilizing cells with 0.25% Triton X-100 prior to fixation with paraformaldehyde, blocking with PBS containing 2% bovine serum albumin (2% PBS-BSA), and 2% donkey serum for 1 h. Cells were subsequently incubated for 1 h with mouse anti-ceramide primary antibody (1:300 in 2% PBS-BSA; Sigma-Aldrich, St. Louis, MO, USA) and 45 min (protected from light) with Alexa Fluor 488 donkey anti-mouse IgG secondary antibody (1:500 in 2% PBS-BSA; Life Technologies, Carlsbad, CA, USA). Representative images shown were collected the Zeiss AxioObserver D1 microscope and objective described above, with exposure and gain settings kept consistent during collection of control and raloxifene-treated images.

### PicoGreen NET Quantification Assay

All incubations were performed at 37°C and 5% CO_2_ unless otherwise noted. Isolated neutrophils were plated on 96-well tissue culture plates at 2 × 10^5^ cells/well. Cells were pretreated with estrogen receptor antagonists (e.g., raloxifene) for 30 min, then incubated an additional 2 h with PMA (25 nM) to induce NET production. Micrococcal nuclease was then added at a final concentration of 500 mU/ml for 10 min to allow digestion of extracellular DNA. Following addition of 5 mM EDTA, plates were centrifuged at 200 × *g* for 8 min; supernatant samples (100 μl) were then collected and transferred to a 96-well plate. DNA was quantified using a Quant-iT PicoGreen^®^ dsDNA Assay Kit from Life Technologies (Carlsbad, CA, USA).

### Quantification of Estrogen Receptor Expression

Freshly isolated neutrophils were harvested and 1 × 10^6^ cells resuspended in TRIzol Reagent (Thermo Fisher Scientific, Waltham, MA, USA). Total RNA was extracted using a Zymo Research Direct-zol RNA kit (Irvine, CA, USA) following the manufacturer’s protocol (including DNAse treatment). Following quantification of RNA using a Thermo Fisher Scientific Nanodrop Spectrophotometer (Waltham, MA, USA), quantitative PCR was performed on an ABI 7000 platform using USB^®^ Veriquest™ Probe One-Step qRT-PCR Master Mix (2×). Predesigned human TaqMan^®^ Gene Expression Assays (Life Technologies, Carlsbad, CA, USA) were used to probe for expression of ESRRG, ESR2, and GAPDH. An equal input of total RNA was used for each assay.

### Transwell Chemotaxis Assay

Neutrophils, pre-incubated for 20 min at 37°C in HBSS alone or HBSS with 10 μM raloxifene, were seeded in 6-mm transwell permeable supports (3-μm pore size; Corning Inc., Corning, NY, USA) that were placed in 24-well plates; lower chambers contained either HBSS alone or 100 nM f-Met-Leu-Phe (fMLP). Following a 45-min incubation at 37°C, inserts were removed, and cells were lysed by addition of Triton X-100 (0.1% final, 10 min, 24°C). To determine the relative levels of migration to the lower well, the colorimetric elastase substrate *N*-methoxysuccinyl-Ala-Ala-Pro-Val *p*-nitroanilide was added to lysed cell samples (10 mM final); after a 30-min incubation at 24°C, absorbance at 405 nm was measured using a SpectraMAX Gemini EM fluorescence reader (Molecular Devices, Sunnyvale, CA, USA).

### Phagocytosis Assay

Following a 30-min incubation at 37°C in the presence or absence of 10 μM raloxifene, neutrophils were combined in a 96-well plate (2 × 10^5^ cells/well) with pHrodo Red *S. aureus* Bioparticles (Life Technologies, Carlsbad, CA, USA) as specified by the manufacturer. Plates were incubated at 37°C, and phagocytosis was assessed by measuring fluorescence intensity (560 nm excitation, 585 nm emission) at 15 min intervals using a SpectraMAX Gemini EM fluorescence reader (Molecular Devices, Sunnyvale, CA, USA).

### ROS Production Assays

Neutrophils were incubated in HBSS supplemented with 10 mM 2′,7′-dichlorofluorescein diacetate (DCFDA) for 20 min at 37°C with gentle agitation. Neutrophils were then centrifuged at 400 × *g* for 5 min, washed with HBSS, and centrifuged again using the same settings before being counted and resuspended in HBSS at a concentration of 5 × 10^6^ cells/ml. Samples (100 μl) of cell suspension were then added to a 96-well plate (5 × 10^5^ cells/well) and incubated with HBSS or raloxifene for 30 min prior to addition of PMA. Fluorescence intensity (485 nm excitation, 530 nm emission) was measured at 15-min intervals using a SpectraMAX Gemini EM fluorescence reader over 2 h; between reads, plates were incubated at 37°C while protected from light.

### Total/Phospho-ERK ELISA

Relative levels of total/phospho-ERK were determined using InstantOne ELISA kits (eBiosciences, San Diego, CA, USA) according to the manufacturer’s protocol, with minor modifications (11). Total/phospho ERK was quantified following PMA stimulation (25 nM) for 45 min in the presence or absence of raloxifene (10 μM).

### NET Killing Assay

Neutrophils suspended in serum-free RPMI (SF-RPMI) were added to 48-well plates at a density of 4 × 10^5^ cells/well. SF-RPMI or raloxifene (10 μM final) was added to applicable wells, and cells were incubated for 30 min at 37°C with 5% CO_2_ (identical wells containing no neutrophils were also prepared). Following addition of PMA to applicable wells, cells were incubated for a further 4 h at 37°C with 5% CO_2_. Overnight cultures of USA300 MRSA (strain UAMS 1182) bacteria were resuspended in RPMI containing 10% 70°C heat-inactivated fetal bovine serum (FBS) to achieve a density of 8 × 10^5^ colony forming units (CFUs)/ml. Fifty microliters of bacterial suspension were added to each well, resulting in a 2% final concentration of FBS and a multiplicity of infection (MOI) of 0.1. Following a centrifugation at 1600 rpm for 5 min, mixed neutrophil/bacterial cultures were incubated for 15 min at 37°C with 5% CO_2_. Samples from each well were then collected, serially diluted in sterile H_2_O, and plated on Todd Hewitt Agar for enumeration of CFUs.

### Statistical Analysis

All statistical analyses described in the figure legends were performed using GraphPad Prism version 7.0.

## Results

### Raloxifene Inhibits PMA-Induced NET Production

**Figure 1:**
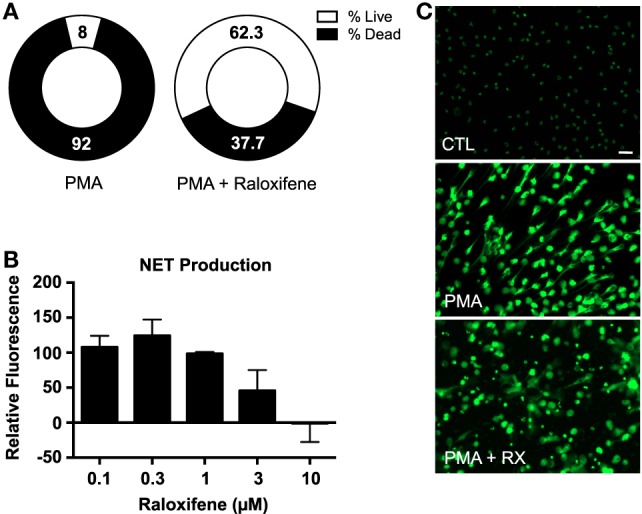
**Raloxifene inhibits PMA-induced NET production**. **(A)** A plate reader-based live–dead screening assay was used to assess the affects of raloxifene on PMA-induced cell death. **(B)** PMA-induced NET production was quantified in cells pre-incubated with raloxifene at the indicated concentrations; PicoGreen DNA dye was used to measure extracellular DNA (NET production expressed as a percent of PMA-only control; *n* = 5). **(C)** Visualization of NET production by control (CTL) or PMA-induced NETs in the presence or absence of raloxifene (3 μM). DNA/NETs stained with Sytox Green. Data shown are expressed as mean values ± SE of the indicated number of biological replicates (each performed in triplicate). Where applicable, results were analyzed by one-way ANOVA.

An image-based cell viability screen (2) using the fluorescent DNA dye Sytox Green identified raloxifene as one of the small molecules from the ~1200 pharmacologically active known compounds tested to have enhanced the survival of neutrophils when exposed to PMA, a potent NET inducer (Figure 1A), suggesting that raloxifene treatment may inhibit NET production. To directly assess the effect of raloxifene on NET production, we quantified release of DNA using the fluorescent DNA dye PicoGreen, finding a concentration-dependent inhibition of PMA-induced NET production by the drug (Figure 1B). These results were confirmed *via* fluorescence microscopy of SYTOX Green-stained cells, which revealed a reduction in the number of NET-positive cells and an increase in the number of intact, condensed nuclei (Figure 1C).

### Raloxifene Does Not Affect Neutrophil Chemotaxis or Phagocytosis but Reduces Intracellular Ceramide

**Figure 2:**
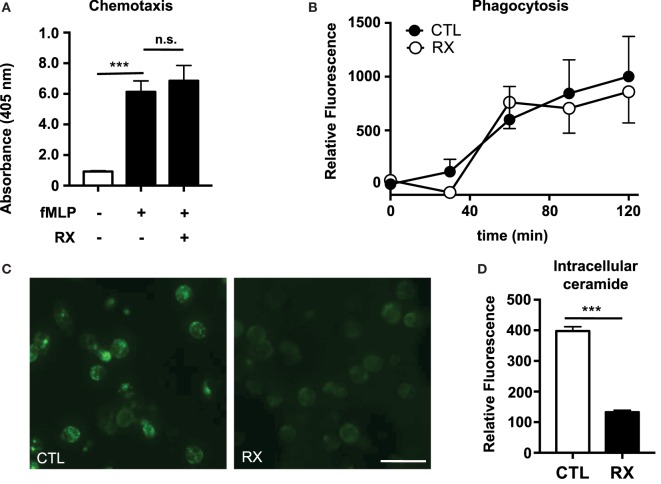
**Raloxifene does not affect neutrophil chemotaxis or phagocytosis but reduces intracellular ceramide**. **(A)** Transwell chemotaxis assays were used to quantify neutrophil migration in a gradient of the chemottractant fMLP (100 nM) in the presence or absence of 10 μM raloxifene (*n* = 3). **(B)** Neutrophils were incubated with pH-sensitive *S. aureus*-labeled bioparticles in the presence or absence of 10 μM raloxifene (*n* = 3). **(C)** An immunostaining approach was used to visualize ceramide accumulation in untreated control (CTL) or raloxifene-treated (RX; 10) neutrophils (45 min incubation); representative images from three separate experiments are shown. **(D)** Mean fluorescence intensity (MFI) values were collected using region of interest analysis to quantify relative ceramide levels in control and 10 μM raloxifene-treated neutrophils. Results shown represent average MFI values for 96–193 cells imaged using identical settings in three separate experiments. Where applicable, results were analyzed by one-way ANOVA and *post-hoc* Dunnett’s test or Student’s *t*-test. ***P < 0.001 vs. control values.

We previously demonstrated that tamoxifen, in addition to stimulating NET production, enhances both chemotaxis in a chemoattractant gradient and phagocytosis of bacteria-labeled bioparticles (4). Tamoxifen treatment also increases intracellular ceramide levels in neutrophils *via* inhibition of glucosylceramide synthase, a key enzyme in the sphingolipid synthesis pathway that converts ceramide to glucosylceramide. Tamoxifen-induced NET production is dependent on this increase of intracellular ceramide (4). In contrast to our observations with tamoxifen, we found that raloxifene had no statistically significant effect on either chemotaxis in a gradient of the chemoattractant fMLP (Figure 2A) or phagocytosis of *S. aureus*-labeled bioparticles (Figure 2B). Furthermore, immunostaining of raloxifene-treated neutrophils revealed that, in contrast to tamoxifen, raloxifene reduces intracellular ceramide levels (Figures 2C,D).

### Selective Estrogen Receptor Antagonists Inhibit PMA-Induced NET Production

**Figure 3:**
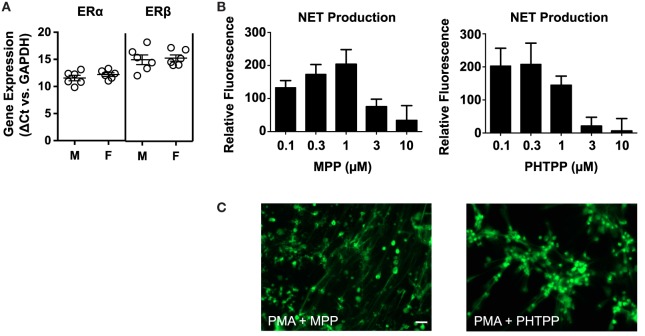
**Selective antagonists of ERα and ERβ inhibit PMA-induced NET production**. **(A)** Quantitative PCR was used to measure ERα and ERβ gene expression in neutrophils from 12 healthy donors [6 males (M), 6 females (F)]. Data expressed as ΔCt values relative to GAPDH. **(B)** PMA-induced NET production was quantified in cells pre-incubated with MPP (selective ERα antagonist) or PHTPP (selective ERβ antagonist) at the indicated concentrations; PicoGreen DNA dye was used to measure extracellular DNA (NET production expressed as a percent of PMA-only control; *n* = 5). **(C)** Visualization of PMA-induced NET production in cells pre-incubated with MPP or PHTPP. DNA/NETs stained with Sytox Green. Data shown are expressed as mean values ± SE of the indicated number of biological replicates (each performed in triplicate). Where applicable, results were analyzed by one-way ANOVA and *post hoc* Newman–Keuls test.

Quantitative PCR-based analysis of ERα and ERβ expression in neutrophils from 12 healthy donors (6 male and 6 female) revealed that circulating neutrophils express both nuclear receptors (Figure 3A). Fluorescence-based quantification of NET production indicated that the selective estrogen receptor antagonists MPP (ERα inhibitor) and PHTPP (ERβ inhibitor) mimicked the raloxifene-mediated inhibition of PMA-induced NET production (Figure 3B). Imaging of SYTOX Green-stained cells revealed some residual NET production by neutrophils pre-incubated with MPP and PHTPP, suggesting the potential involvement of multiple estrogen receptors in raloxifene-mediated inhibition of NETosis (Figure 3C).

### Raloxifene Inhibits PMA-Induced ERK Phosphorylation but Not PMA-Induced ROS Production

**Figure 4:**
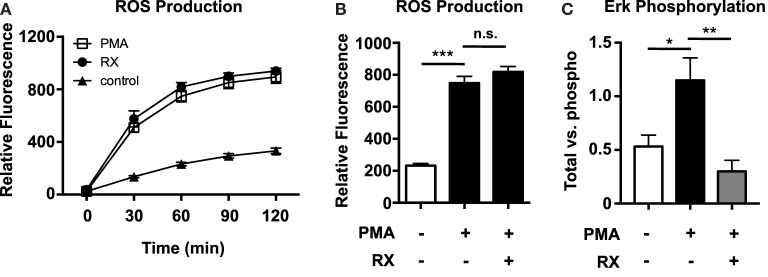
**Raloxifene inhibits PMA-induced ERK phosphorylation, but not PMA-induced ROS production**. ROS production in control and 25 nM PMA-treated neutrophils in the presence/absence of raloxifene (10 μM) was quantified over time **(A)** and at 2 h **(B)** using the cell-permeable fluorescent ROS probe DCFDA (*n* = 3). **(C)** PMA-induced ERK phosphorylation in the presence/absence of 10 μM raloxifene was quantified using total/phospho ELISAs (*n* = 4). Data shown are expressed as mean values ± SE of the indicated number of biological replicates (each performed in duplicate or triplicate). Where applicable, results were analyzed by one-way ANOVA and *post hoc* Newman–Keuls test. *P < 0.05, ***P < 0.001 vs. control values.

Reactive oxygen species production was quantified using the membrane-permeable fluorescent ROS probe 2′,7′-dichlorofluorescein diacetate (DCFDA). Although PMA-induced NET production is known to be ROS dependent (1), raloxifene did not inhibit PMA-induced ROS production (Figures 4A,B). However, ELISA-based quantification of ERK phosphorylation revealed that raloxifene significantly inhibited PMA-induced ERK activation (Figure 4C), a critical signaling event that has been shown to play a role in NET production (2).

### Raloxifene Inhibits NET-Mediated Killing of Methicillin-Resistant *Staphylococcus aureus*

**Figure 5:**
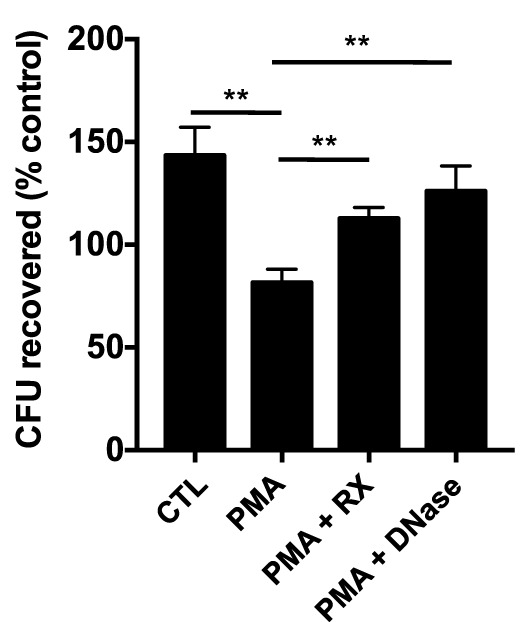
**Raloxifene inhibits NET-mediated killing of methicillin-resistant *Staphylococcus aureus***. Neutrophil killing of USA300 MRSA was quantified in a NET-based killing assay. Control (CTL) represents bacteria exposed to unstimulated neutrophils and PMA represents neutrophils treated with 25 nM PMA. Raloxifene (RX; 10 μM) or DNase (7.5 U/ml) were added 30 min prior or 3.5 h following addition of PMA, respectively (*n* = 3). Data shown are expressed as mean values ± SE of three independent experiments, each performed in triplicate. Results were analyzed by Student’s *t*-test. **P < 0.01 vs. control values.

To determine whether raloxifene affects NET-based bactericidal activity of neutrophils, we performed a NET-based killing assay in which neutrophils were pre-stimulated with PMA to induce NET production prior to challenge with methicillin-resistant *Staphylococcus aureus* (MRSA; MOI 0.1). Stimulation of NETs *via* 4 h incubation with 25 nM PMA produced a statistically significant reduction in bacterial survival in neutrophil/MRSA cocultures (Figure 5). Incubation with raloxifene 30 min prior to addition of PMA reversed this effect.

## Discussion/Conclusion

Here, we show evidence that the SERM raloxifene inhibits PMA-induced NET production. Unlike tamoxifen, an estrogen receptor modulator that induces NET production (4), raloxifene reduces intracellular ceramide levels. Neutrophils from both male and female donors express the nuclear estrogen receptors ERα and ERβ, the best characterized targets of raloxifene. Selective antagonists of either receptor inhibited PMA-induced NETosis, suggesting that raloxifene may act through multiple receptors to inhibit NET production, and further indicating that tamoxifen-induced NET production is largely dependent on estrogen receptor-independent, non-specific effects (e.g., inhibition of glucosylceramide synthase). We find that raloxifene inhibits PMA-induced ERK phosphorylation, a signal transduction event known to be critical for NETosis (2); thus, raloxifene likely modulates NET production *via* regulation of ERK signaling.

Our results suggest that raloxifene may have beneficial therapeutic effects in contexts where excessive NETosis is undesirable and associated inflammatory changes cause damage to the host (12). NETs appear to play a role in the pathophysiology of rheumatoid arthritis (13); notably, raloxifene has been shown to exhibit significant antiarthritic properties in a murine model of this disease (14). The incidence of rheumatoid arthritis in raloxifene-treated postmenopausal women (and specifically the contribution of NETs to disease pathophysiology) represents an important area for further investigation; however, our results also suggest that raloxifene therapy may come at some consequence to patients at high risk of infection, as raloxifene inhibits NET-mediated bacterial killing.

These findings provide new insight into the effects of raloxifene on neutrophil function, which at present are poorly understood. Such effects hold important clinical implications, given the large number of patients receiving this drug. Further exploration of the pathways mediating the anti-NETosis activity of raloxifene may reveal new therapeutic targets and facilitate development of therapies that can more selectively regulate NET production in appropriate physiological contexts.

## Ethics Statement

All studies described here were reviewed and approved by the local (UCSD) Institutional Review Board.

## Author Contributions

RC and VN conceptualized and led the project. RC, RF, SD, CS, and AH designed and performed experiments and interpreted data. RC, RF, and VN wrote the manuscript.

## Conflict of Interest Statement

The authors declare that the research was conducted in the absence of any commercial or financial relationships that could be construed as a potential conflict of interest.
